# Fix and forget or fix and report: a qualitative study of tensions at the front line of incident reporting

**DOI:** 10.1136/bmjqs-2014-003279

**Published:** 2015-03-06

**Authors:** Tanya Anne Hewitt, Samia Chreim

**Affiliations:** 1 Department of Population Health, University of Ottawa, Ottawa, Ontario, Canada; 2 Telfer School of Management, University of Ottawa, Ottawa, Ontario, Canada

**Keywords:** Incident reporting, Near miss, Patient safety, Qualitative research, Attitudes

## Abstract

**Introduction:**

Practitioners frequently encounter safety problems that they themselves can resolve on the spot. We ask: when faced with such a problem, do practitioners fix it in the moment and forget about it, or do they fix it in the moment and report it? We consider factors underlying these two approaches.

**Methods:**

We used a qualitative case study design employing in-depth interviews with 40 healthcare practitioners in a tertiary care hospital in Ontario, Canada. We conducted a thematic analysis, and compared the findings with the literature.

**Results:**

‘Fixing and forgetting’ was the main choice that most practitioners made in situations where they faced problems that they themselves could resolve. These situations included (A) handling near misses, which were seen as unworthy of reporting since they did not result in actual harm to the patient, (B) prioritising solving individual patients’ safety problems, which were viewed as unique or one-time events and (C) encountering re-occurring safety problems, which were framed as inevitable, routine events. In only a few instances was ‘fixing and reporting’ mentioned as a way that the providers dealt with problems that they could resolve.

**Conclusions:**

We found that generally healthcare providers do not prioritise reporting if a safety problem is fixed. We argue that fixing and forgetting patient safety problems encountered may not serve patient safety as well as fixing and reporting. The latter approach aligns with recent calls for patient safety to be more preventive. We consider implications for practice.

## Introduction

Voluntary incident reporting systems have been recommended by various bodies as a way to improve patient safety.^1–4^ Yet, incident reporting systems are complex sociotechnical systems^5^ ^6^ that have come under intense scrutiny;^7–11^ they have been criticised^12–14^ and praised.^15–17^ One of the main objectives of incident reporting systems is to attain organisational learning.^1^ ^18^ This learning is restricted if only realised incidents are reported, but it could be greatly enhanced if patient safety hazards (defined as conditions that could lead to patient harm) are reported as well.^19^ ^20^ While many studies have researched obstacles to and enablers of incident reporting by front-line healthcare workers, this study focuses on a specific but significant type of information—problems that the practitioners themselves can typically resolve. Few studies have looked at the goal conflict associated with the decision made at the front end of fixing an encountered patient safety problem on the spot and forgetting about it, or fixing the problem and reporting it into a reporting system. For example, a practitioner is about to administer medication to a patient, when s/he realises that the dosage by far exceeds what is recommended. The practitioner has two options: (1) seek clarification and change the dosage, administer the proper medication, move on to other tasks, and forego incident reporting, or (2) seek clarification and change the dosage, administer the proper medication, and fill out an incident report.

Several conceptualisations of what constitutes an incident have been suggested.^21–23^ The Canadian Patient Safety Institute (CPSI) presents three types of incidents: a harmful incident (reached the patient, and harm resulted—typically well accepted in healthcare under various terms such as ‘adverse events’, ‘sentinel events’ and ‘critical incidents’) (ref. 24, p.9), a no-harm incident (reached the patient, but no discernible harm resulted) and a near miss (did not reach the patient). Given that all of these deserve analysis, it can be argued that they should be reported into a reporting system. The WHO's Patient Safety Curriculum Guide expects multidisciplinary learners to acquire competencies in adverse events and near misses.^25^ Franklin *et al* note that “If incident reporting systems include and encourage reports of no-harm incidents in addition to actual patient harm, they can facilitate monitoring the resilience of healthcare processes” (ref. 19, p.765). The Health Foundation's The Measurement and Monitoring of Safety states “The focus is moving from counting harms after the event towards looking at hazards that might give rise to error, or safety failure before harm has occurred” (ref. 26, p.iii). Overall, while historically adverse events were the main focus of hospital reporting systems, near misses and hazards may also be expected in patient safety reporting, thus requiring practitioners to attend to this dimension of their work.

At a cognitive level, Tucker and Edmondson^27–29^ studied healthcare practitioners’ first order problem solving (fixing the problem at hand) and second order problem solving (understanding why the problem exists, aiming to correct the drivers, and thereby enhancing organisational learning). This study extends Tucker and Edmondson's work by viewing their concepts as applied to a reporting system, specifically first order problem solving represented as fixing a safety problem in the moment and forgetting about it, and second order problem solving represented as fixing the problem in the moment and reporting it so that wider learning can occur. The choice between these two options may be done more or less purposefully, as we demonstrate in this paper. Regardless, the two options are faced on a regular basis, and are underpinned by competing organisational priorities that require providing effective, expedient care to as many patients who need that care, and investing effort to create awareness of hazards and incidents. Given that front-line workers tend to be in an excellent position to identify safety problems and that they can play an important role in enhancing organisational learning,^28^ we ask: How do front-line healthcare practitioners choose between (A) fixing a patient safety problem and forgetting about it, and (B) fixing the problem and reporting it into an incident reporting system?

## Methods

This study is part of a larger research project on voluntary incident reporting and safety in a teaching hospital in Ontario, Canada. The reporting system at the hospital is available to employees through any networked device. The reporter enters information using the patient's medical record number and provides a narrative describing the patient safety incident (see the CPSI definition above) using facts. The reports are reviewed by Clinical Managers who investigate them locally, by physician Clinical Reviewers who assess if harm was caused to the patient, and by Core Reviewers who assess larger hospital issues.

Our study focused on General Internal Medicine—one of the largest departments in the hospital. The study began in spring 2012, with a quality review meeting whereby the researchers were introduced to key personnel who would later become interviewees. Over 5 months, two researchers (independently and together) confidentially interviewed hospital personnel. The Chief Physician and Clinical Director were key informants, each of whom recommended other personnel to interview based on our request to sample individuals with a variety of views and practices related to incident reporting. This process yielded 26 interviews purposefully sampled. Additional interviewees were recruited through email requests for practitioners to participate in the study. Overall, 40 participants were recruited as shown in table 1.

**Table 1:** Number of interviewees by job title

Job title	Number of participants
Clinical Director	1
Physicians (Chief Physician, Clinical Observer, clinical reviewers, physicians)	8
Residents	3
Clinical managers, nurse educators	4
Registered nurses, registered practical nurses	15
Clerks, orderlies	3
Physiotherapists	3
Pharmacy (pharmacist, drug distribution supervisor, pharmacy technician)	3
Total	40

The interview included questions about reporting, non-reporting and safety practices. Interviews averaged 45 min, and were digitally recorded and transcribed. Data analysis was undertaken by two researchers who met to discuss the themes in the interviews and the derivation of codes based on the data gathered. Atlas ti software (GmbH, Berlin, Germany) was used to code the interviews and retrieve quotations. The analysis involved a deductive and inductive approach.^30^ Through a reading of the literature, we were informed about concepts and approaches related to incident reporting systems and patient safety (deductive approach). Our analysis of the data revealed local practices related to the use of the incident reporting system (inductive approach). Through an iterative process of moving between the literature and the data, we identified three themes pertaining to how patient safety problems that healthcare practitioners can solve are handled with respect to reporting.

## Results

The Canadian tertiary care hospital where this voluntary incident reporting system was in place had instructions on the types of events expected to be entered: a ‘patient safety event’ is ‘Any circumstance where a patient experiences harm, or potential harm, due to medical care’ (Hospital literature). This is a broad statement; leaders refined and defined expectations regarding incident reporting, but these expectations were not always met by practitioners. A nursing leader described two different ways that events might be dealt with:

We had dry alcohol swabs…So nurses would go ‘oh dry, dry, dry’, they'd go through 5–6 dry ones, get a wet one, and move on with their day. Finally somebody comes to me and says ‘why are all of our alcohol swabs coming up dry?’… So it takes that initiative. Some nurses have that initiative … they think about systems. Others think about moments, ‘this is an issue right now, this is something I can deal with…’ not ‘oh I think the hospital needs to know that this isn't working’. (*Educator 1*)

In the situation where the nurse opens alcohol swabs that are dry, and continues to do so until finding a wet one, the nurse is ‘fixing and forgetting’. The goal of getting a wet alcohol swab was reached—the nurse fixed the problem—so work can continue. However, the nurse who questions why there were dry swabs is ‘fixing and reporting’. The nurse fixed the problem—getting the wet alcohol swab as well—but ensured that the problem was reported before attending to the next task. The report allowed an investigation and went beyond only tending to the immediate problem at hand. This illustrates a situation that healthcare practitioners can address, followed by their choice to report or not to report, which is the subject of our study. We examine the context surrounding practitioners’ decision to ‘fix and forget’ or ‘fix and report’. Through this analysis, we revealed three themes: handling near misses, fixing individual patients’ safety problems and adapting to imperfections.

### Handling near misses

Near misses have various definitions,^23^ ^24^ but in all these definitions, the incident has not been realised—some intervention prevented the near miss from progressing to a harmful incident. Some near misses, if reported, would overwhelm the practitioners:

You have a crazy shift… Somebody has turned around and quickly they grab an IV fluid, they go to hang it, they realize it's in their hand and say ‘oh it's the wrong one’ and they go put it back. I basically have been told that that's a near miss, you should be doing an incident report. But we would drown in paperwork if we did that, right? (*Registered Nurse 9*)

The main issue here was that reporting this type of near miss would create overburdening paperwork, and the subtext of the patient not being harmed helped justify the decision to ‘fix and forget’. In this case, putting the incorrect intravenous fluid back and getting the right intravenous fluid for the patient's need, and continuing on with various tasks was prioritised, without reporting the near miss. However, near misses were not reported for other reasons as well.

If the physician wrote in the wrong chart they [nurses] will just call the physician up and say ‘hey you wrote the order in the wrong chart come and write it in the right chart’…They wouldn't fill out an incident report unless something had happened because of an order being written wrong… If they had a chance to fix it, it's not considered a near miss. Like I said, most near misses are not reported… there's no agreement on what constitutes a near miss. (*Educator 2*)

What constituted a near miss was poorly understood, and there was general agreement that they were under-reported. Generally, the view was that if a problem had not progressed to the patient (CPSI near miss), there would be no need to report. The potential for harm to the patient notwithstanding, the patient was not harmed, so ‘fixing and forgetting’ as opposed to ‘fixing and reporting’ was how near misses were generally handled.

However, some did recognise that near misses were worth reporting:

(*Interviewer*) Would you fill out [an incident report] for a dose that's a hundred times too high on the prescription and you got it clarified down to what it should have been?

(*Registered Practical Nurse 2*) I should, yeah, that would be a near miss type thing, because it never got given but it had the potential to be harmful.

This nurse considered that the potential for harm was a trigger to writing an incident report, and in contrast to his/her colleagues, engaged in ‘fixing and reporting’.

### Fixing individual patients’ safety problems

Healthcare providers prioritise caring for individual patients, and if a problem occurs, providers tend to treat the situation as ‘a one-off’, or ‘a one-time’ event. Hazards are conditions or situations that could cause harm, but if the patient was not harmed, the situation was seen as not worth reporting. A prescription or order was ambiguous, but instead of viewing this as a problem worthy of reporting, the nurse got clarification so that the proper drugs were administered to his/her patient, and did not subsequently fill an incident report.

Anything that adversely affects my patient is an incident report … If there's a potential, it becomes more of a judgement thing … ‘If we can fix it, don't report it’ type idea… A doctor writes an order, I don't understand what they've [written], I call the doctor and say ‘what was the dosage…’ They tell me. It hasn't affected my patient …. If there wasn't any effect on the patient—I fixed the problem—so I don't do an incident report. (*Registered Nurse 9*)

This quote demonstrates two concepts—severity determining reporting, as well as responsibility towards an assigned patient. This nurse was certain that an incident that affects the patient is worthy of reporting (a CPSI harmful incident), but if a situation is fixed, the severity would decrease, and there would be no need to report. Additionally, the quote illustrates the responsibility felt towards assigned patients (‘my’ patient) and the desire to fix problems for individuals under a provider's direct care. An incident report would have little effect on an individual patient, so ‘fixing and forgetting’ was seen as far more aligned with what the healthcare provider believed to be his or her role. In a different scenario, where a patient was affected (a CPSI no harm incident), an incident report was not thought to be important.

[The nurses] called me [and] we fixed the situation—we checked [the patient's] blood sugars and everything was fine … Because the mistake was recognized early and because the appropriate recourses were taken, it wasn't something that needed to be reported. (*Resident 2*)

In this case, short acting insulin had been injected instead of the intended long acting insulin, but the healthcare team realised it early enough to prevent any serious harm to the patient. The reactive response of the healthcare providers was considered appropriate, and ‘everything was fine’ for this patient; an incident report was seen not to be necessary. This is in contrast to one interviewee, who noted:

[We should report] having two patients side by side with the exact same last name—which I have seen multiple times—you're asking for error to happen. (Physician 2)

This physician talked of a hazard that can affect more patients than just an individual patient assigned to a practitioner as being worthy of reporting since the possibility of causing an incident is foreseeable. Reporting a hazard aligns with the hospital's expectation of reporting, ‘A circumstance where a patient experiences potential harm due to medical care’ (hospital literature). This is a case that is closer to ‘fixing and reporting’. It should be noted, however, that most interviewees espoused the ‘fix and forget’ option and only a few spoke about reporting hazards.

### Adapting to imperfections

Fixes, or adapting to unfixed problems, can become routinised normal work, and may not be noticed any longer. The extract below followed a discussion that only major events would be reported.

(*Interviewer*) So [a patient whose paperwork wasn't complete], staying for the extra 2 days of the weekend isn't major?

(*Physician 3*) Happens all the time. There's delays. My day is chock a block full of dealing with these sort of things as well as trying to take care of patients and do other duties… They're just things that occur day-to-day, that have always sort of occurred day-to-day in various different ways. That is part of the practice of medicine in a big large teaching hospital.

The sense of inevitability of ‘these sorts of things’ is evident in stating the daily occurrence of seemingly minor problems that this practitioner needed to deal with to attend to patients and other duties. Reporting these problems, although identified as potentially harmful to patients, was not undertaken—was not considered. Rather, practitioners adapted to these seemingly minor issues and considered these as routine occurrences.

(*Resident 1*) [Requisitions] get lost all the time and that's never reported.

(*Interviewer*) And it's not reported?

(*Resident 1*) Oh no. Everybody agrees that there's probably some black box …where all these radiology reqs that have been lost—that happens all the time… They say ‘oh well we never got the fax’… And that never gets reported, and it delayed treatment or delayed assessment.

Here, although the potential for harm was acknowledged by the provider, the problem was not reported—rather the resident (and other practitioners) adapted to the frequent loss of requisitions and delayed assessments. Engaging in workarounds, such as hand delivering a requisition, became a routinised practice that escaped attention as a problem to be reported.

In short, ‘fixing and forgetting’ was the main choice that most practitioners made in situations where they faced problems that they themselves could resolve. These situations included (A) handling near misses, which were seen as unworthy of reporting since they did not result in actual harm to the patient, (B) prioritising solving individual patients’ safety problems, which were viewed as unique or one-time events, and (C) encountering re-occurring safety problems, which were framed as inevitable, routine events.

## Discussion

This study looked at specific encounters with hazards and problems where a healthcare practitioner might either fix the problem and move on, or fix the problem and report it into the reporting system. We identified three themes: handling near misses, fixing individual patients’ safety problems and adapting to imperfections. In these scenarios, the practitioners nearly always chose to fix and forget—or to engage in first order problem solving. It is worth reflecting here on Tucker and Edmondson's study, where they found that “on average, 33 min were lost per 8 h shift due to coping with system failures that could have been addressed and removed” (ref. 27, p.60). These different types of problems that practitioners fix and forget are lost to organisational learning, and may be costing them time as well in their workarounds. Choosing to be efficient in the moment may ironically cause the front-line providers to be far less efficient over time than intended.

One of the problems was near misses. Near misses in our study were poorly understood—a common occurrence in other healthcare contexts. Kessels-Habraken *et al*^23^ argued that healthcare has focused more on incidents that did reach the patient but did not cause harm (CPSI no harm incident—the insulin example in this study) than incidents that did not reach the patient (CPSI near miss—ambiguous prescription example in this study). Kessels-Habraken *et al* indicate that due to this focus, “valuable safety-related information about successful error recovery mechanisms remains unavailable or gets lost” (ref. 23, p.1302). Other healthcare studies have shown near misses not being tapped for their potential. Mattioli *et al*^31^ noted a preference for reporting incidents causing harm over near miss reporting in a paediatric surgical department. Jeffs *et al*^32^ also found that near misses generate three typical responses: doing a quick fix and nothing else (‘fixing and forgetting’ in our study), which was the most frequent response, and two types of ‘fixing and reporting’, whereby the report falls into a black hole, or is used as a catalyst for organisational change. In transportation, accident investigation crews examine crash sites extensively to find out why the crash occurred and how to prevent it in the future. They thus generate lessons for the industry, but only after lives have been lost in the crash. Alternately, near misses are seen as ‘free lessons’ where learning can occur, but without any deaths.^33^ Burnett, Carthey and Vincent stated that high-risk industries have shifted from focusing attention on incidents and realisation of harm to hazards and conditions that create safety.^26^ In healthcare, Schildmeijer *et al* recommend that random chart reviews be undertaken to find no-harm incidents,^34^ although this view is not unanimous.^35^ Franklin *et al* (ref. 19, p.770) state that “The challenge, across all areas of harm, is now to create and use data on… low-harm occurrences to test the resilience of safety practices and systems”.

Fixing an individual patient's safety problem is common. As mentioned, healthcare providers often personalise the assignment of patients to them, and the care given (including fixing problems) to ‘my’ patient is then a source of professional pride and responsibility. Jeffs *et al* noted a physician stating “part of what we pride ourselves in is to be able to get ourselves out of tricky situations” (ref. 32, p.289). This professional pride in fixing problems is common to many front-line workers, including in rail^36^ and nursing.^28^ Practitioners view their ability to solve problems ‘… as a strong sign of their expertise and competence’ (ref. 37, p.101). Fixing problems that would otherwise harm that patient (to the exclusion of reporting the hazard) is common. However, as Jeffs *et al* (ref. 32, p.289) point out “As a result of such unreported quick-fix scenarios, learning remained local and confined to the individual level. Notably, learning is limited to the individual who initiated the fix.” Haradan adds (ref. 38, p.2) “We fix it for that particular person or family and the immediate surgical team involved learns, which is very important, but what about preventing such errors from happening to anyone else?”. Tucker and Edmondson^27^ note that nurses are encouraged to use vigilance to solve problems to the detriment of organisational improvement. This aligns directly with first order problem solving (fixing and forgetting), to the exclusion of second order problem solving (fixing and reporting).^27–29^ In fixing and reporting, the lessons are disseminated beyond the local circumstance of the individual patient, and can benefit far more patients as a result.

It is worth noting that opting to report or not to report is not always a purposeful decision. For example, if fixing and forgetting becomes the norm, adapting to imperfections will follow, and the need to report hazards or problems will escape attention. Waring writes “the inevitability of error leads to more than acceptance [of errors], but also to their ‘normalisation’. This is where some common mistakes are regarded as routine and normal within the context of medical work, and in consequence these events are not perceived as problematic or worth reporting” (ref. 6, pp.1931–1932). Dekker explains Vaughan's influential term coined through her study of the Challenger launch decision: “The ‘normalisation of deviance’ describes a process whereby a group's construction of risk can persist even in the face of continued (and worsening) signals of potential danger… Small departures from an earlier established norm are often not worth remarking or reporting on” (ref. 39, p.538). Thinking of imperfections as routine occurrences could result in hazards or safety problems never being reported.

However, not all healthcare practitioners are blind to the imperfections. Tucker and Edmondson describe a desirable employee as a “disruptive questioner who will not leave well enough alone. The person is constantly questioning, rather than accepting and committed to, current practices” (ref. 27, p.68). The success of the Central Line Blood Stream Infection initiative in the USA^40^—an excellent example of a patient safety intervention—began with a physician challenging the status quo, or the inevitability of error. Reporting problems can save front-line healthcare workers from encountering the same incident repeatedly, or engaging in workarounds that reinforce working with an imperfect system.^27^ Even in our study, a nurse saw the potential for patient harm through an order clarification, and a physician found two patients with the same last name in close proximity troubling, and both deemed the situation worthy of fixing and reporting.

At this point, one might ask whether it is worth reporting all realised incidents and all potential incidents. Might this not result in costly and far too many reports, for the reporters,^41^ and those who analyse the reports?^20^ ^42^ And more importantly, would such reporting yield safety benefits? For example, it has been pointed out that most events that are entered into reporting systems, such as patient falls, ‘provide little incremental value about the insight of safety systems… questioning the benefit of having the user report them in the first place’ (ref. 20, p.155). In contrast, other studies^43^ have looked at patient falls with an aim to get more of them reported. Importantly, most studies suggest that better criteria should be set to guide practitioners about what and how to report. Additionally, ‘operational ‘know how’ and conceptual ‘know why’’ are important for front-line engagement in quality improvement projects (ref. 20, p.125). Certainly the most serious harm to patients is dealt with expediently using hospital processes of risk management and senior personnel,^42–44^ often followed by extensive investigations. Equally important, however, is giving consideration to reporting near misses and hazards, and using them as the focus of investigation and intervention. This would enable investigations to have a preventive (and not only a reactive) approach to patient safety.^44^

### Limitations

This study has limitations. The focus on the fixable types of problems is to the exclusion of other types of reports that healthcare professionals write. Further, this study was undertaken in one hospital department, and may not be generalisable to other departments. However, the results concerning the under-reporting of near miss incidents have been found in other studies of hospital departments, notably paediatric surgery,^31^ paediatric ICU,^45^ radiation oncology,^46^ ^47^ emergency^48^ and surgery.^49^ ^50^ In addition, it should be noted that the strength of qualitative studies lies not in generalisability, but in the ability to provide an in-depth view of micro dynamics present in sites.^51^ In fact, findings from qualitative studies can be transferred to other contexts that are similar to the one studied.^30^ The quotes and interpretation should serve to illuminate the context sufficiently to allow the reader to assess if the findings of this study are transferable to other contexts.

## Conclusion

This study investigated a particular type of possible report—a problem that the practitioners themselves can typically resolve—and looked at their choice between ‘fixing and forgetting’ and ‘fixing and reporting’. In considering the themes of handling near misses, fixing individual patients’ safety problems, and adapting to imperfections, we found that generally healthcare providers do not prioritise reporting if a situation is fixed. This suggests a number of practice recommendations. ‘Fixing and forgetting’ could engender normalisation as exceptions become the norm, but recognition of hazards and problems may help healthcare guard against normalising deviance, and may help improve patient safety by focusing on a more preventive approach. Communication and training on what should be reported, why and how should be considered more carefully. Further, recognising the ‘disruptive questioner’^27^ as a desirable employee would be one step towards encouraging this new approach. Finding individuals who ‘fix and report’, enabling them to share their reporting approach with their colleagues, and recognising their efforts would underlie informal training that could result in more healthcare workers fixing and reporting hazards and problems, and help organisational learning that improves patient safety.
